# Production of human blood group B antigen epitope conjugated protein in *Escherichia coli* and utilization of the adsorption blood group B antibody

**DOI:** 10.1186/s12934-016-0538-z

**Published:** 2016-08-11

**Authors:** Wenjing Shang, Yafei Zhai, Zhongrui Ma, Gongjin Yang, Yan Ding, Donglei Han, Jiang Li, Houcheng Zhang, Jun Liu, Peng George Wang, Xian-wei Liu, Min Chen

**Affiliations:** 1 The State Key Laboratory of Microbial Technology, National Glycoengineering Research Center, School of Life Sciences and Shandong Provincial Key Laboratory of Carbohydrate Chemistry and Glycobiology, Shandong University, Jinan, Shandong 250100 People’s Republic of China; 2 The Institute of Medical Molecular Genetics, Department of Biochemistry and Molecular Biology, Bin Zhou Medical University, No. 346, Guan Hai Road, Lai Shan District, Yan Tai City, Shan Dong Province 264003 People’s Republic of China

**Keywords:** Immunoadsorption, Blood group B antigen, Conjugated glycoprotein, *E. coli* O-antigen, PglB

## Abstract

**Background:**

In the process of ABO-incompatible (ABOi) organ transplantation, removal of anti-A and/or B antibodies from blood plasma is a promising method to overcome hyperacute rejection and allograft loss caused by the immune response between anti-A and/or B antibodies and the A and/or B antigens in the recipient. Although there are commercial columns to do this work, the application is still limited because of the high production cost.

**Results:**

In this study, the PglB glycosylation pathway from *Campylobacter jejuni* was exploited to produce glycoprotein conjugated with *Escherichia coli* O86:B7 O-antigen, which bears the blood group B antigen epitope to absorb blood group B antibody in blood. The titers of blood group B antibody were reduced to a safe level without changing the clotting function of plasma after glycoprotein absorption of B antibodies in the plasma.

**Conclusions:**

We developed a feasible strategy for the specific adsorption/removal of blood group antibodies. This method will be useful in ABOi organ transplantation and universal blood transfusion.

**Electronic supplementary material:**

The online version of this article (doi:10.1186/s12934-016-0538-z) contains supplementary material, which is available to authorized users.

## Background

The ABO blood group system is the most important blood type system in humans. Blood type incompatibility means the exposure of A or B antigen to a person who has antibodies against these antigens [1]. These antibodies act as haemagglutinins, which cause blood cells to clump and break apart, and can even cause death when large amounts of such cells are encountered after transfusion or organ transplant. Removal of anti-A and/or B antibodies from plasma is a promising method to overcome hyperacute rejection and allograft loss [2]. Several protocols have been employed to remove antibodies or antibody-producing cells in the process of ABOi organ transplantation [3], among which immunoadsorption has attracted more attention because of its specificity. The most commonly used immunoadsorbers are glycosorb columns with A/B blood group antigens linked to a sepharosematrix [4, 5]. Unfortunately, A and B blood group antigens are difficult to acquire and immobilize [6].

At present, most A/B antigens used in glycosorb columns are synthesized by chemical methods or enzymatic synthesis. One of the most difficult steps in the chemical synthesis of well-defined oligosaccharide antigens is the stereospecific formation of glycosidic linkages between monosaccharide units [7]. Enzymatic synthesis utilizing the corresponding glycosyltransferase is limited by the availability of enzymes and the cost of activated sugar donors [8]. Accordingly, it is necessary to find a low-cost and highly-effective method to produce A/B antigens to remove anti-A/B antibodies from plasma.

**Fig. 1:**
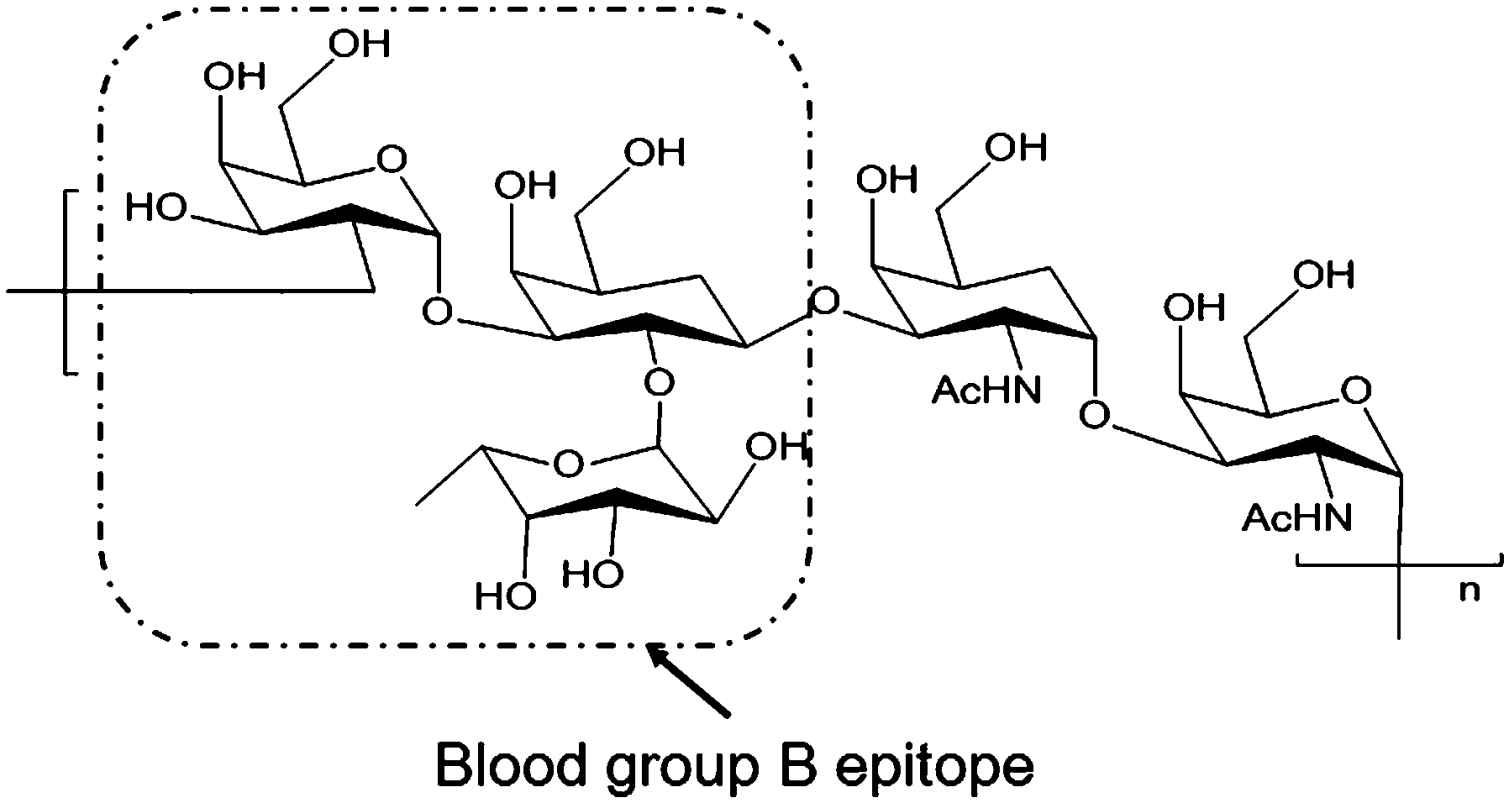
The O-antigen repeat unit structure of *E. coli* O86:B7. The human blood group B antigen epitope is labeled in a *dashed box*

The O-antigen in *Escherichia coli* (*E. coli*) O86:B7 has been shown to possess high human blood group B activity because of the structural similarity between the O-antigen and human blood group B antigen epitope [9–11] (Fig. 1). Therefore, *E. coli* O86:B7 can be a potential cell factory of B antigens. We plan to obtain a type of glycoprotein loaded with this O-antigen that can be used to remove the A/B antibody from plasma. The oligosaccharyl transferase PglB from *Campylobacter jejuni* (*C. jejuni*) can transfer a wide range of polysaccharides from undecaprenyl-pyrophosphate (Und-PP) linked precursors to the asparagine of the consensus sequence D/E-X-N-Y-S/T (X, Y ≠ Proline) of the carrier protein in the periplasm [12, 13]. The *N*-glycosylation pathway from *C. jejuni* in recombinant *E. coli* has been shown to be a simple method for producing glycoprotein [12].

**Fig. 2:**
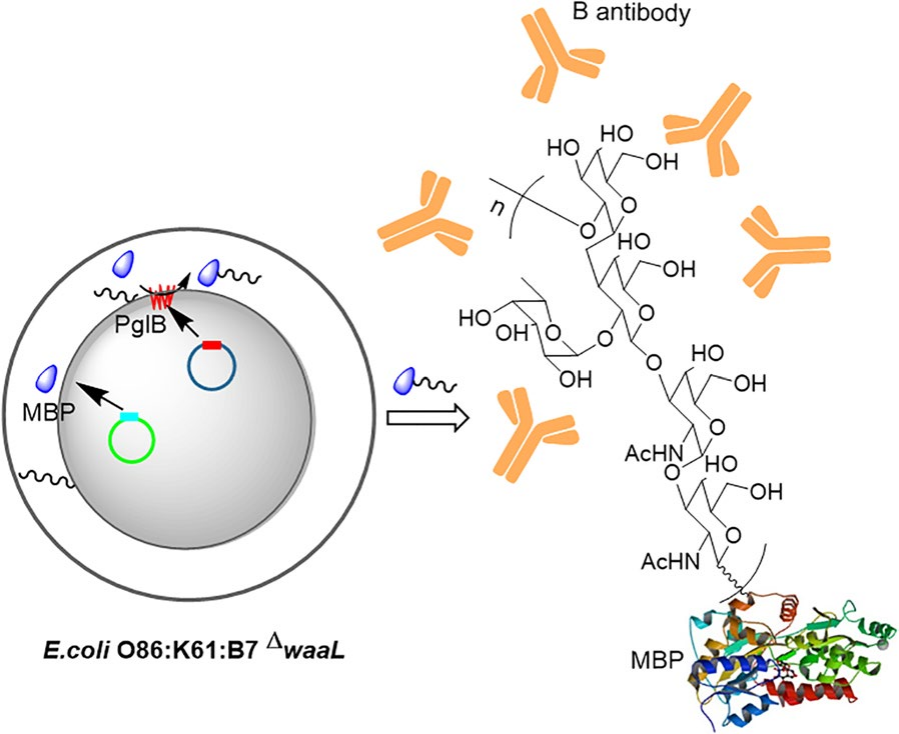
The scheme of the production of MBP_mut_-OPS and its application

In our work, the *N*-glycosylation pathway of *C. jejuni* was used to produce glycoprotein conjugated with the O86 O-antigen (Fig. 2). The O86 O-antigen conjugated-protein could adsorb anti-B antibody in the plasma, and the parameters of coagulation were not affected after the adsorbing process. Furthermore, it would have potential use in universal blood transfusion and may also be used in ABOi organ transplantation.

## Results and discussion

### The production and detection of MBP_mut_-OPS (O86:B7) bioconjugates

To obtain glycoprotein loaded with OPS of O86:B7, PglB from *C. jejuni* was cloned into *E. coli* O86:B7 to transfer the O-antigen onto the protein, resulting in a kind of glycoprotein with OPS.

**Fig. 3:**
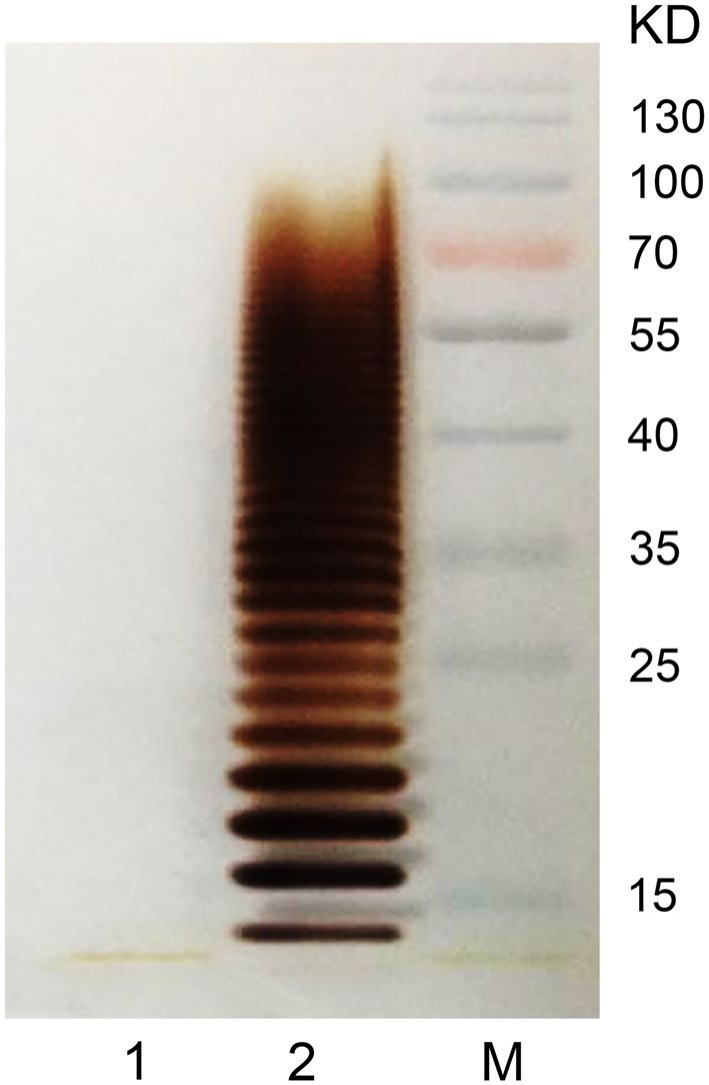
Silver staining result of LPS. The silver staining was detected on 12 % gel. Line 1: LPS extracted from *E. coli* O86Δ*waaL*; Line 2: LPS extracted from *E. coli* O86 wild type; M: protein Marker

In *E. coli,* the OPS is transferred to lipid A by the WaaL enzyme to produce LPS. To effectively conjugate the OPS on a protein by PglB, the *waaL* gene was deleted from *E. coli* O86 using a λ-Red recombination system. The *waaL* gene deletion was confirmed using test primer pair t-*waaL*-F/t-*waaL*-R, which could amplify across the deletion area. Furthermore, ladder straps were observed on the SDS-PAGE gel of LPS from wild *E. coli* O86, while no straps were observed in the lane of the LPS extracted from *E. coli* O86 Δ*waaL*, which indicated that the LPS of *E. coli* O86 Δ*waaL* had a low degree of polymerization (Fig. 3). Therefore, we successfully obtained a strain of *E. coli* O86:B7 without the *waaL* gene.

**Fig. 4:**
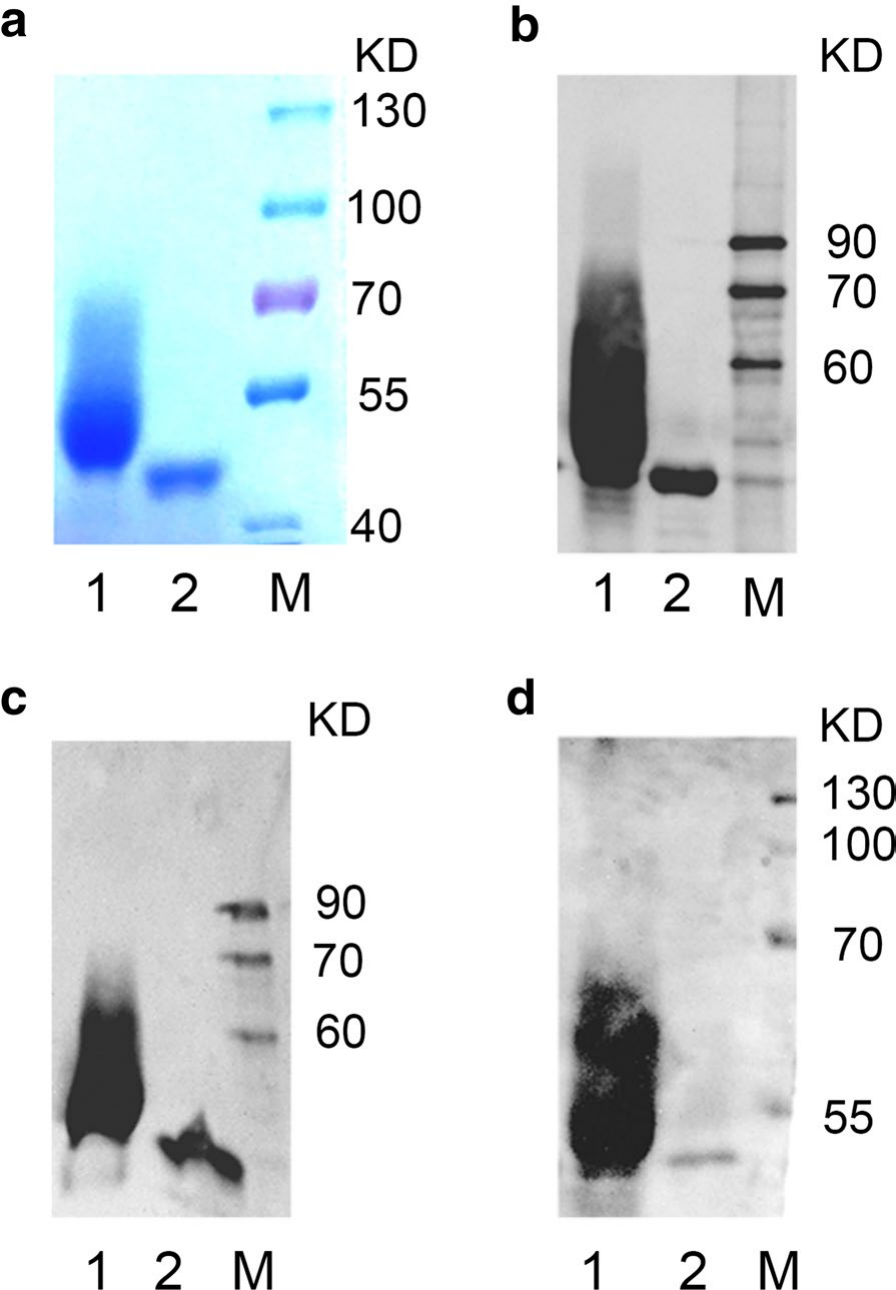
SDS-PAGE and western blot analysis of MBP_mut_ and MBP_mut_-OPS from *E. coli* O86:B7. SDS-PAGE analysis was carried out on 8 % gel (**a**). Western blot was detected by 8 % gel using anti-His antibody (**b**), anti-MBP antibody(**c**) and anti-O86 antibody (**d**), respectively. Line 1: purified MBP_mut_-OPS from *E. coli* O86:B7; Line 2: purified MBP_mut_; M: protein Marker

Maltose-binding protein (MBP) was selected as a carrier protein for OPS with B antigen activity. MBP is expressed in the periplasm of *E. coli* by the *malE* gene [14], which is generally used as a tag for expression and purification of foreign recombinant proteins [15] with a Amylose-Resin column. MBP without an *N*-glycosylation site was modified to MBP_mut_ with four consensus sequences at the C terminal for loading with blood group B antigen epitope by cloned PglB in *E. coli* O86 Δ*waaL*. The glycosylated MBP_mut_ (i.e. MBP_mut_-OPS) and unglycosylated MBP_mut_ were purified from *E. coli* O86 Δ*waaL* with or without the plasmid pACT3-*PglB* and identified by SDS-PAGE (Fig. 4a) and western blot using anti-His antibody (Fig. 4b), anti-MBP antibody (Fig. 4c) and anti-O86 OPS antibody (Fig. 4d), respectively.

When probed using the anti-His and anti-MBP antibody, a ladder of bands appeared on the blot when MBP_mut_ was expressed in *E. coli* O86 Δ*waaL* with PglB (Fig. 4 lane 1), indicating that the protein was conjugated with multi-units of OPS (MBP_mut_-OPS), while unglycosylated MBP_mut_ expressed in *E. coli* O86 Δ*waaL* without PglB showed only one band with a molecular weight of 44 kDa, as expected (Fig. 4 lane 2). During the process of western blot, O86 antiserum instead of monoclonal anti-O86 antibody was used to determine the O-antigen activity of MBP_mut_-OPS. Therefore, nonspecific reaction might occur in form of the lightgray band on the western blot. These results were consistent with the finding that OPS had typical variability of chain length with different degrees of polymerization [16]. Furthermore, conjugation of OPS on MBP_mut_ was confirmed by MALDI-TOF mass spectrometry. As expected, the molecular weight of MBP_mut_-OPS (47,898.93 Da) was higher than that of MBP_mut_ (44,017.39 Da) (Additional file 1: Figure S1). The average number of OPS repeat units in purified MBP_mut_-OPS was four based on the molecular weight of one repeat unit of OPS (894 Da) and MBP_mut_ (44017 Da). Moreover, 1.5 mg MBP_mut_-OPS was purified from 1 L of fermentation, which provided a way to obtain a large yield of glycoproteins using *E. coli* O86. We believe that the yield can be improved after optimizations such as culture conditions, fermentation method. Still, the cost of this approach to produce B-antigen absorption material is much lower than tradition method which includes enzymatic synthesis of B-antigen saccharide using the corresponding glycosyltransferases because of the limited availability of enzymes, the high cost of activated sugar donors, etc.

### Ability of MBP_mut_-OPS conjugates to bind to blood group B antibody

**Fig. 5:**
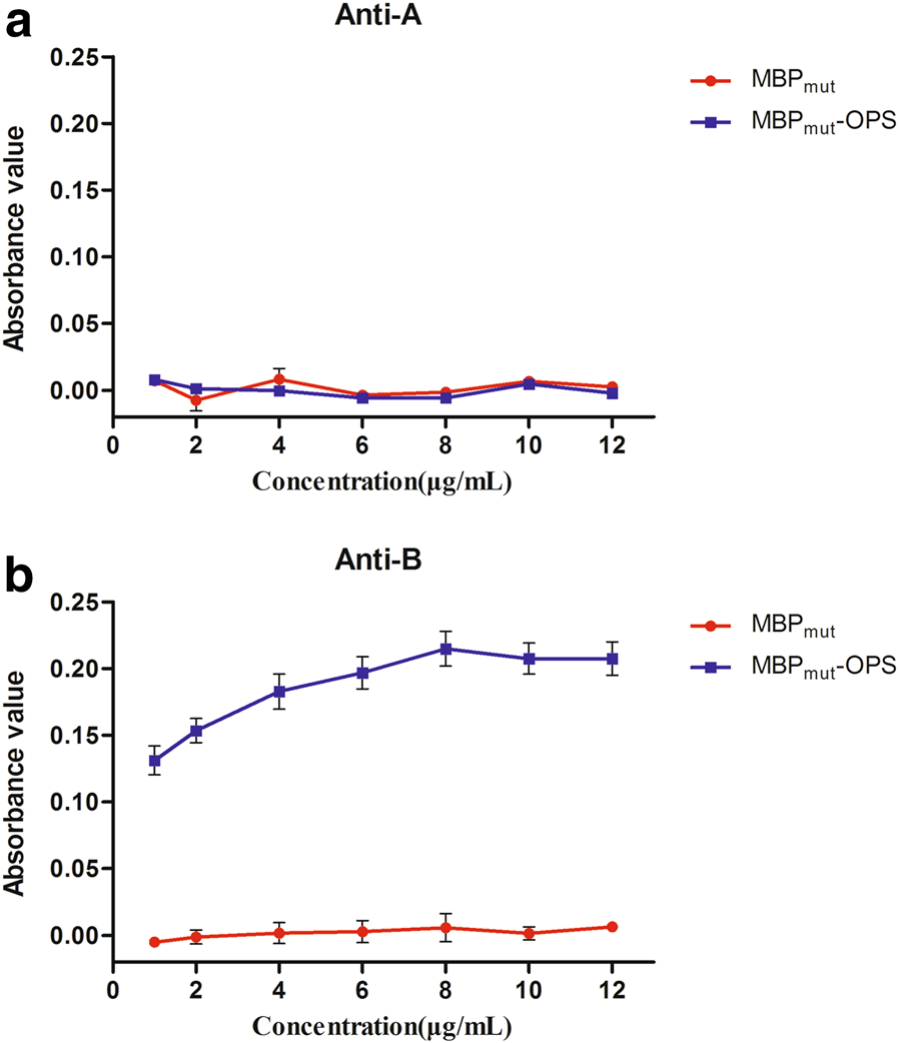
Binding of anti-B antibody to MBP_mut_-OPS from *E. coli* O86:B7. Antibody binding was assessed by ELISA in duplicate

An ELISA assay was conducted to measure the ability of MBP_mut_-OPS conjugates to bind with anti-A/B antibody. Unglycosylated MBP_mut_ without blood group activity was used as a negative control. The MBP_mut_-OPS can be recognized by the anti-B antibody (Fig. 5b), but not by the anti-A antibody (Fig. 5a). No binding between unglycosylated MBP_mut_ and anti-A/B antibody was detected. These results suggested that MBP_mut_-OPS could bind anti-B antibody.

### Specific absorption of blood group B antibody in the plasma

**Fig. 6:**
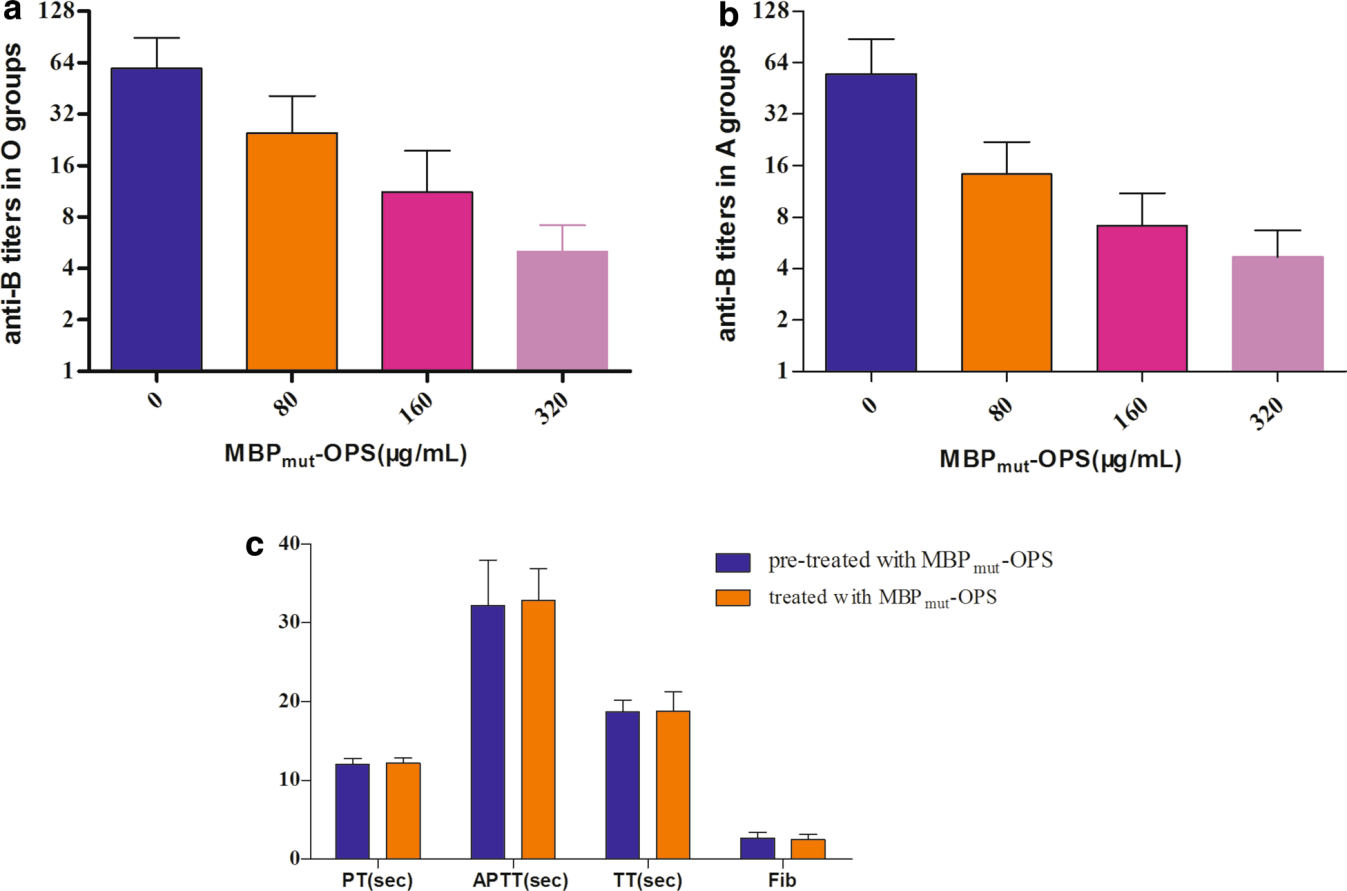
The titers of anti-B antibodies and clotting parameters in the plasma before and after adsorption with MBP_mut_-OPS. The titers of anti-B antibodies in plasma samples of blood group O (**a**) and blood group A (**b**) before and after adsorption with different amount of MBP_mut_-OPS were measured. The clotting parameters in plasma treated/pre-treated with MBP_mut_-OPS were detected with fully automatic blood coagulation analyzer (**c**). The hollow columns present the values of untreated plasma samples, while the filled ones denote the results of absorbed plasma samples. *Error bars* represent the standard deviation from three duplicates

Based on the results of ELISA, MBP_mut_-OPS was applied to remove anti-B antibodies from blood group O and group A plasma as a B antigen. The removal rates of B antibody in plasma increased as increasing amounts of glycosylated MBP_mut_ were added. The average blood group anti-B antibody titer of plasma samples of blood group O decreased from 64 to 4 (Fig. 6a) after treatment with MBP_mut_-OPS (320 μg/mL). A previous report indicated that the antibody titer of plasma samples ≤8 is compliant with the restricting final titer for undergoing surgery [17]. Analogously, upon evaluation of the plasma of blood group A, the titers of all samples decreased to a safe level of 4 after adsorption with a final concentration of 160 μg/mL MBP_mut_-OPS (Fig. 6b).

To investigate the effects of MBP_mut_-OPS on blood coagulation, the parameters of PT, APTT, TT, and Fib were detected within 4 h of blood withdrawn to citrate. None of the above parameters in the treated sample with MBP_mut_-OPS differed significantly from the control and levels remained normal (Fig. 6c). These results demonstrated that MBP_mut_-OPS could absorb anti-B antibody effectively and did not affect the coagulation properties of the plasma. Thus, the purified glycoprotein with blood group B epitope has great potential for clinical applications. MBP with O-antigen of *E. coli* O86 could be used to remove anti-B antibody from group O or A in emergency transfusions without strict matches. The produced MBP_mut_-OPS in *E. coli* O86 Δ*waaL* will contribute significantly to the development of a method for universal blood transfusion. Furthermore, for actual clinical applications, the endotoxin of the glycoprotein produced from *E. coli* cells should be removed to a safety level, which will be taken into consideration in our following study.

## Conclusions

This study successfully glycosylated MBP with B antigen using a novel one-shot approach. In the system, large quantities of glycoproteins are produced and have great potential for further clinical development in many fields including ABOi organ transplantation and universal blood transfusion. In addition, glycoprotein with blood group antigens could also be used as research tools or alternative drugs for infection or other diseases associated with blood group antigens. A similar strategy could extend to blood group A antigen since anti-A agglutinins were reported to be absorbed by an A active *E. freundii* [18].

## Methods

### Bacterial strains, plasmids and growth condition

*Escherichia coli* O86:B7 (ATCC 12701) was obtained from American Type Culture Collection (Rockville, MD). Commercially available IgM monoclonal anti-B antibody (obtained from clone HEB-29) was purchased from Merck Millipore (Billerica, USA). All strains and plasmids used in this study were listed in Additional file 1: Table S1. All strains were grown in Luria–Bertani broth (LB) at 37 °C. *E. coli* DH5α and O86 were used for plasmids cloning and glycoprotein expression experiments, respectively. Ampicillin (100 μg/mL), 50 μg/mL kanamycin and 34 μg/mL chloramphenicol were added to the media for selection as needed. Plasmids pKD4, pKD46 and pCP20 were used for the deletion of the gene coding for the O-polysaccharide ligase *WaaL* of *E. coli* O86. Plasmid pACT3 and pBAD24 were used for the expression of PglB and MBP protein, respectively.

### Knockout of *waaL* gene of *E. coli* O86

The *waaL* gene of *E. coli* O86 was knocked out to obtain O86 Δ*waaL*: FRT using λ-Red recombination system. Briefly, using plasmid pKD4 as template, the kanamycin-resistant gene flanked by homologues of *waaL* gene was amplified by PCR with knockout primers. When induced by *L*-arabinose, plasmid pKD46 could express three recombinant proteins (Exo, Beta, Gam) of λ-prophage, which assisted the replacement of *waaL* gene with kanamycin-resistant gene. Subsequently, the kanamycin-resistant gene was eliminated by FLP-promoted recombination system using plasmid pCP20 and the *E. coli* O86 Δ*waaL* was obtained successfully. The knockout primers (k-*waaL*-F, k-*waaL*-R) and test primers (t-*waaL*-F, t-*waaL*-R) used in the knockout experiments were listed in Additional file 1: Table S1. The extraction of LPS was carried out according to the instruction of LPS extraction kit (iNtRON Biotechnology, KOREA). The silver staining experiment was performed as reported previously [19].

### Construction of recombinant plasmids

In order to ensure the successful glycosylation of MBP by PglB, the consensus sequence D-Q-N-A-T was repeated four times and inserted at the C terminal of MBP. Overlap PCR was used to amplify the *malE*_mut_ gene with primers *malE*-F, *malE*-R1, *malE*-R2 and *malE*-R3 (Additional file 1: Table S1). Restriction sites for *Sal* I *and Hin*d III at their 5′ ends of primers were used for the insertion of the modified gene into the vector pBAD24, and thus the plasmid pBAD24-*malE*_mut_ was obtained with a 6× His tag (i.e. N-HHHHHH-C) between *Sma* I and *Sal* I of pBAD24 (Induced by L-arabinose). Likewise, the *pglB* gene from *C. jejuni* NCTC 11168 was inserted between *Sma* I and *Sal* I of plasmid pACT3 (Induced by IPTG) and the plasmid pACT3-*PglB* was obtained.

### Glycoprotein expression and purification

The recombinant plasmids pBAD24-*malE*_mut_ and pACT3-*PglB* were co-transformed into *E. coli* O86 Δ*waaL* to obtain an engineering strain with the ability to produce MBP_mut_-OPS bioconjugates. Plasmid containing MBP_*mut*_ gene was transformed into *E. coli* O86 Δ*waaL* to produce unglycosylated MBP_mut_ as a control. *E. coli* O86 Δ*waaL* transferred with pBAD24-*malE*_mut_ and pACT3-*PglB* was grown in 50 mL LB broth at 37 °C for 16 h, with shaking. Cultures were then inoculated 1/100 into 1 L TB broth and further grown at 37 °C with shaking until OD_600_ reached 0.6. Subsequently, 0.1 % (w/v) *L*-arabinose and 50 μM IPTG were added to induce the expression of MBP and PglB, respectively. After further incubation at 28 °C for 6 h, 0.1 % (w/v) *L*-arabinose was added again for continuous induction of MBP.

After that, cells were pelleted by centrifugation at 10,000 rpm for 15 min at 4 °C, and then resuspended in lysis buffer (50 mM PBS, 200 mM NaCl, 5 % glycerin, pH 7.4). The supernanant of cells after ultrasonic lysates was purified using pre-equilibrated Ni-nitrilotriacetic acid (NTA) columns under native conditions. Washing buffer (50 mM PBS, 200 mM NaCl, 5 % glycerin, 50 mM imidazole, and pH 7.4) and elution buffer (50 mM PBS, 200 mM NaCl, 5 % glycerin, 250 mM imidazole, and pH 7.4) were sequentially used. Fraction containing the purified glycoconjugate was collected and then desalted using centrifugal filter (Amicon^®^ Ultra-15, Milipore) against PBS (PH 7.4). The concentration of the proteins was measured with Bradford method.

### Detection of purified glycoprotein

Western blotting was used to detect MBP and MBP_mut_-OPS expression. Samples were separated on 8 % SDS-denatured polyacrylamide gel and were then transferred onto nitrocellulose membrane. Membranes were blocked in 3 % BSA solution for 1 h at room temperature, and then were incubated with anti-hexahistine (anti-His) monoclonal antibody and anti-MBP monoclonal antibody (Beyotime Biotechnology, China), as well as anti-O86 O-antigen polyclonal antibody (Tianjin Biochip Corporation, China), respectively overnight at 4 °C. The secondary antibodies with a horseradish peroxidase (HRP) (Abcam, UK) were used subsequently. The image acquisition was finished by Flour ChemQ (Proteinsimple, US). MALDI-TOF result was analyzed by the MALDI-TOF mass spectrometer (AXIMA Confidence, SHIMAZU, Japan) with sinapic acid as the matrix (50 % ACN, 50 % H_2_O, 0.1 % TFA).

### Binding ability measurement of glycoprotein and anti-B antibody

Polystyrene microtiter plates were coated by the purified proteins MBP/MBP_mut_-OPS from *E. coli* O86 at different concentration overnight at 4 °C. The plates were blocked with 2 % BSA in PBS buffer for 2 h at room temperature. After being washed three times with PBST (PBS, 0.05 % Tween-20), the plates were incubated with anti-B antibody diluted to 1:20 for 2 h, or with anti-A antibody (1:20) as control. After washing, the secondary antibody goat anti-mouse IgM conjugated to HRP (1:20,000) (Abcam, UK) was added and maintained for 1 h. Finally, the TMB substrate was used to develop the signal and 1 M HCl was used to terminate the reaction, and the OD was measured at 450 nm on Bio-Rad680 microplate reader (Hercules, California, USA).

### Detection of the B antibody titer and coagulation parameters in the plasma

All blood samples, from 36 healthy people, were collected with citrate anticoagulation tubes, mixing, and were centrifuged at 1000*g* for 10 min to separate plasma. The plasma was divided two portions, one for the detection of B antibody titers, the other for coagulation analysis.

The B antibody titers in the plasma were measured with the polybrene test according to the instruction (Baso Biological Technology Corporation, Zhuhai, China). Briefly, twofold serial dilutions of plasma sample from 1:2 were made with normal saline for each tube. The same volume of 2 % type Bred blood cells were added to each tube and mixed thoroughly. Low ionic medium, polybrene reagent and resuspending were added subsequently and operated based on the instruction, and the smallest dilution which could still agglutinate erythrocyte was determined as the endpoint, and its reciprocal was considered as the titer of the sample plasma.

In order to detect the effects of proteins MBP_mut_-OPS on blood clotting function, coagulation parameters of the samples treated/pre-treated with MBP_mut_-OPS were measured with fully automatic blood coagulation analyzer ACL7000 (BECKMAN, USA).

### Adsorption of blood group B antibody in the plasma

Aliquots of plasma samples of 800 μL were mixed with final concentration of 0, 80, 160 and 320 μg/mL MBP_mut_-OPS, respectively. After incubation at room temperature for 1 h, the B antibody titer and clotting parameters in the plasma were detected as above methods.

### Statistical analysis

The statistical analyses and figures were generated by GRAPHPAD PRISM software version 5.0. Data were shown as mean ± standard deviation (SD). The difference between two groups was compared by *t* test. For multiple comparisons, One-way ANOVA was used. A probability (*P*) value ≤ 0.05 was considered statistically significant.

## Additional file

10.1186/s12934-016-0538-z List of constructed plasmids, strains and primers used in the study. **Figure S1.** MALDI-TOF detection of MBP_mut_ (a) and MBP_mut_-OPS (b).

## Abbreviations

ABOi: ABO-incompatible
MBP: maltose binding protein
OPS: O-polysaccharides
LPS: lipopolysaccharide
P: prothrombin time
APTT: activated partial thromboplastin time
TT: thrombin time
Fib: fibrinogen
