# Supplementary material for: Production of human blood group B antigen epitope conjugated protein in Escherichia coli and utilization of the adsorption blood group B antibody
Source: Microb Cell Fact. 2016 Aug 11;15:138. doi: 10.1186/s12934-016-0538-z (PMC4982269; doi:10.1186/s12934-016-0538-z)
Supplement: Supplementary file 1 — 10.1186/s12934-016-0538-z List of constructed plasmids, strains and primers used in the study. Figure S1. MALDI-TOF detection of MBPmut (a) and MBPmut-OPS (b). [file 12934_2016_538_MOESM1_ESM.docx]

**Additional files**

for

**Production of human blood group B antigen epitope conjugated protein in *Escherichia coli*** [[**and utilization of**](http://microbialcellfactories.biomedcentral.com/articles/10.1186/s12934-016-0449-z) **the adsorption blood group B antibody**](http://microbialcellfactories.biomedcentral.com/articles/10.1186/s12934-016-0449-z)

Wenjing Shang^1,2^, Yafei Zhai^1^, Zhongrui Ma^1^, Gongjin Yang^1^, Yan Ding^1^, Donglei Han^1,^, Jiang Li^1^, Houcheng Zhang^1^, Jun Liu^1^，Peng George Wang^1^, Xian-wei Liu^1,^*，Min Chen^1,^*

*^1^The State Key laboratory of Microbial Technology, National Glycoengineering Research Center，School of Life Sciences and Shandong Provincial Key Laboratory of Carbohydrate Chemistry and Glycobiology, Shandong University,Jinan, Shandong 250100, People’s Republic of China*

*^2^The Institute of Medical Molecular Genetics, Department of Biochemistry and Molecular Biology, Bin Zhou Medical University, No.346, Guan Hai Road, Lai Shan District, Yan Tai City, Shan Dong Province, 264003 PR China.*

*Corresponding author: [chenmin@sdu.edu.cn](mailto:chenmin@sdu.edu.cn)(Min Chen) and xianweiliu@sdu.edu.cn (Xian-wei Liu)

**Contents**

**Table S1.** List of constructed plasmids, strains and primers used in the study

**Figure S1.** MALDI-TOF detection of MBP_mut_ (a) and MBP_mut_-OPS (b)

**Table S1** List of constructed plasmids, strains and primers used in the study

| **PCR primers** |  |  |
| --- | --- | --- |
| k-*waaL*-F: | CTCGAGAAAAAAAACTGGATAGCGTACTGGAACAGAGCTGTGTAGGCTGGAGCTGCTTC |  |
| k-*waaL*-R: | TTACTTGTTTTTCATCGCTAATAATAAGCCGGCGTAAACATGGGAATTAGCCATGGTCC |  |
| t-*waaL*-F: | GTATGTCTCTTGCAGATTTG |  |
| t-*waaL*-R: | ATGGCGTAACTCAAAGATTC |  |
| *PglB*-F: | CACGCCCGGGATGTTGAAAAAAGAGTATTTAAAAAACCC |  |
| *PglB*-R: | CTGGTCGACTCAATGATGATGATGATGATGAATTTTAAGTTTAAAAACTTTAG |  |
| *malE*-F: | ACGCGTCGACAGGAGGCATAGATTATGAAAATAAAAACAGGTGC |  |
| *malE*-R1: | GTCGCGTTCTGATCGCCGCCGGTCGCGTTCTGATCTTCCAGCTGCGCGTCTTTCAGGGC |  |
| *malE*-R2: | GCGTTCTGATCTCCTCCAGTGGCGTTCTGATCGCCGCCGGTCGCGTTCTGATCGCCGCC |  |
| *malE*-R3: | CCCAAGCTTGGTTAATGATGATGATGATGATGGGTCGCGTTCTGATCTCCTCCAGTG |  |
| **Plasmids** |  | **Source** |
| pBAD | araBAD promoter, pBR322 replicon, Ampr | *[*[*1*](#_ENREF_1)*]* |
| pBAD-MBP_mut_ | pBAD24+malEmut(SalⅠ/HindⅢ), Ampr | This study |
| pACT3 | Tac promoter, pACYC184 replicon, Cmr | *[*[*2*](#_ENREF_2)*]* |
| pACT3-PglB | pACT3+PglB (SmaⅠ/SalⅠ), Cmr | This study |
| **Strains** |  |  |
| *E.coli* Top10 | Wild type | EMDBio- sciences, Inc |
| *E.coli* O86:K61:B7 | Wild type | American Type Culture Collection |
| *E.coli O86:B7ΔwaaL* | deletion of waal in E.coliO86:B7 | This study |
| *E.coli O86:B7ΔwaaL* MBP_mut_ | E.coli O86:B7Δwaal containing pBAD-MBPmut | This study |
| *E.coli O86:B7ΔwaaL* MBP_mut_PglB | E.coli O86:B7Δwaal containing pBAD-MBPmutand pACT3-PglB | This study |

^a^The underlined sequences denote the recognition sites of the restriction enzymes.

**Fig. S1.**


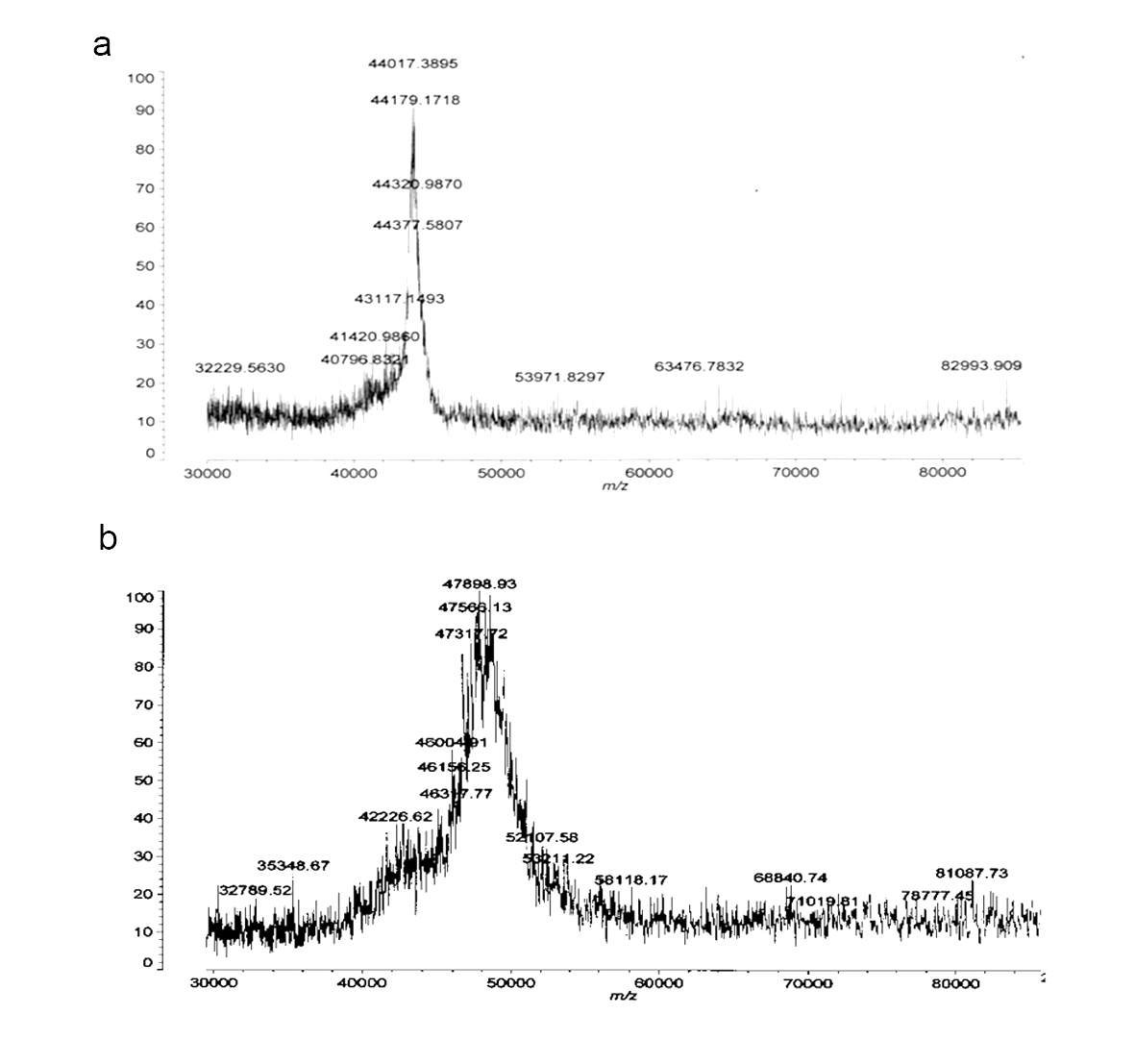


MALDI-TOF detection of MBP_mut_ (a) and MBP_mut_-OPS (b)

**References**

1. Guzman LM, Belin D, Carson MJ, Beckwith J: **Tight regulation, modulation, and high-level expression by vectors containing the arabinose PBAD promoter.** *J Bacteriol* 1995, **177:**4121-4130.

2. Dykxhoorn DM, St Pierre R, Linn T: **A set of compatible tac promoter expression vectors.** *Gene* 1996, **177:**133-136.
